# Supplementary figures and images for: Fortetropin supplementation prevents the rise in circulating myostatin but not disuse-induced muscle atrophy in young men with limb immobilization: A randomized controlled trial
Source: PLoS One. 2023 May 23;18(5):e0286222. doi: 10.1371/journal.pone.0286222 (PMC10204970; doi:10.1371/journal.pone.0286222)

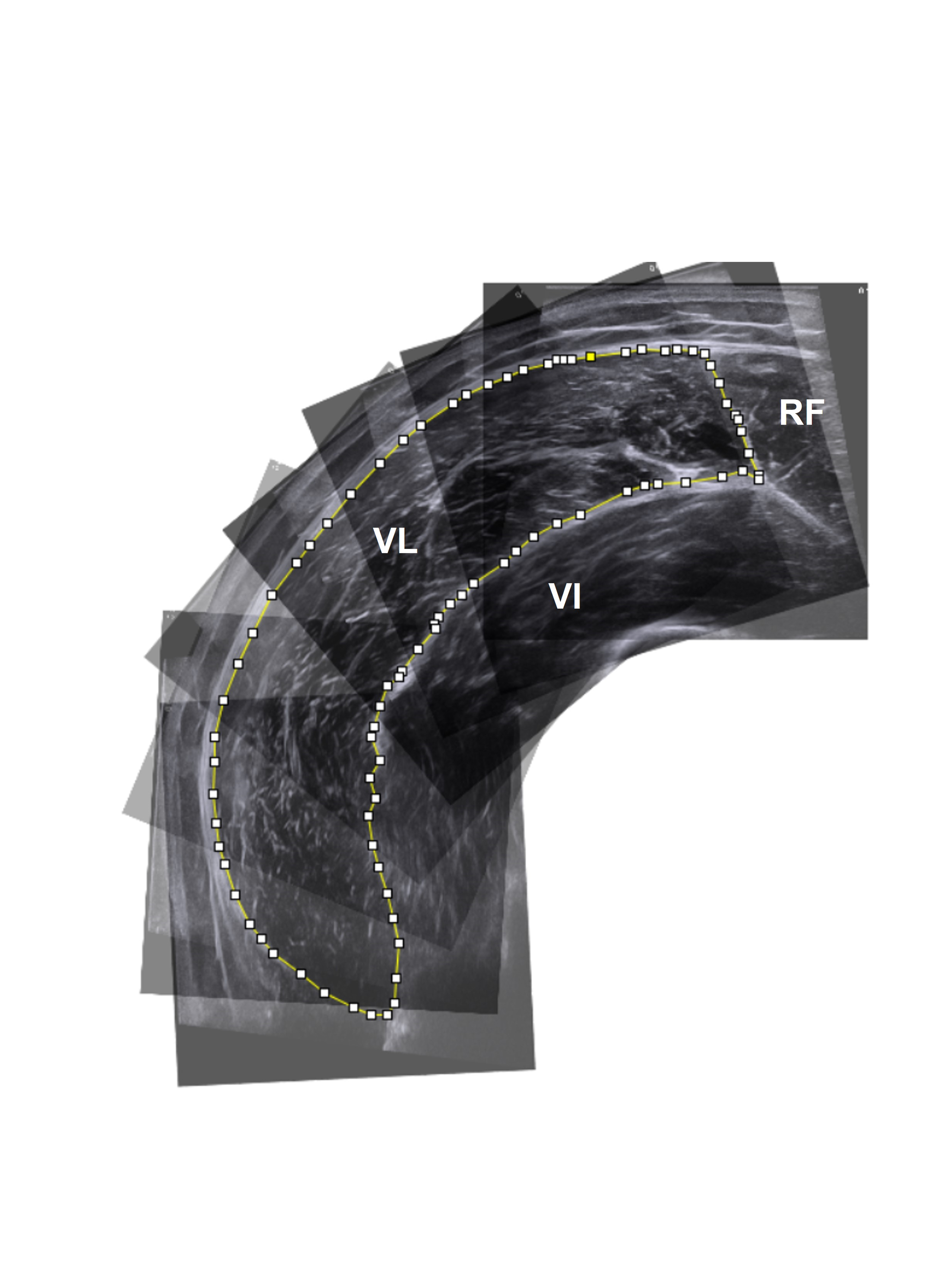

Supplement: S1 Fig — VL, vastus lateralis; VI, vastus intermedius, RF, rectus femoris. (TIF) [file pone.0286222.s001.tif]

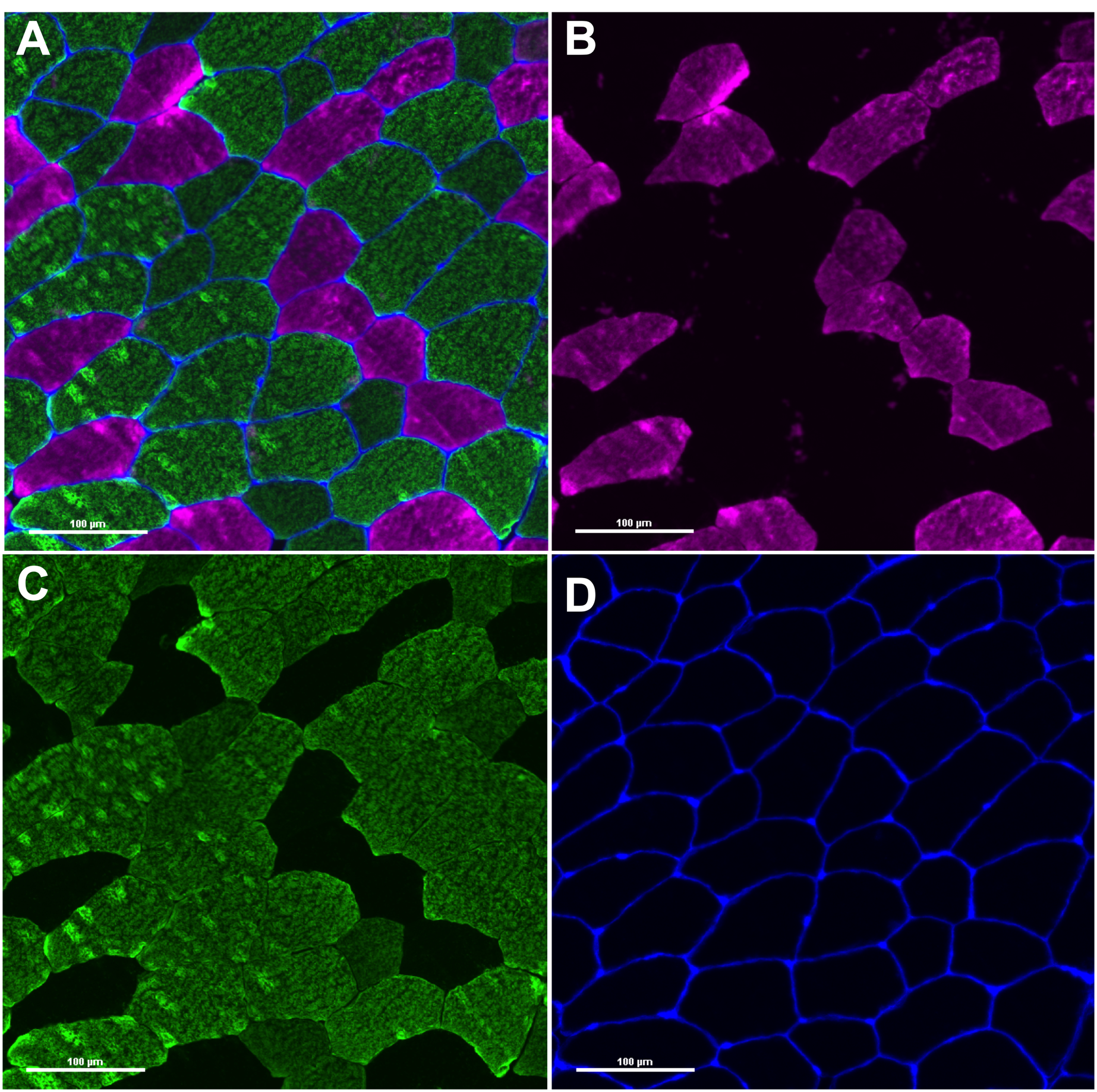

Supplement: S2 Fig — (A) merge of all the panels; (B) Type I fibers, purple; (C) Type II fibers, green; (D) Laminin, blue. (TIF) [file pone.0286222.s002.tif]
