# Supplementary material for: Fortetropin supplementation prevents the rise in circulating myostatin but not disuse-induced muscle atrophy in young men with limb immobilization: A randomized controlled trial
Source: PLoS One. 2023 May 23;18(5):e0286222. doi: 10.1371/journal.pone.0286222 (PMC10204970; doi:10.1371/journal.pone.0286222)
Supplement: S1 File — FOR-SUPP, Fortetropin® supplement; PLA-SUPP, placebo supplement. (DOC) [file pone.0286222.s003.doc]

**
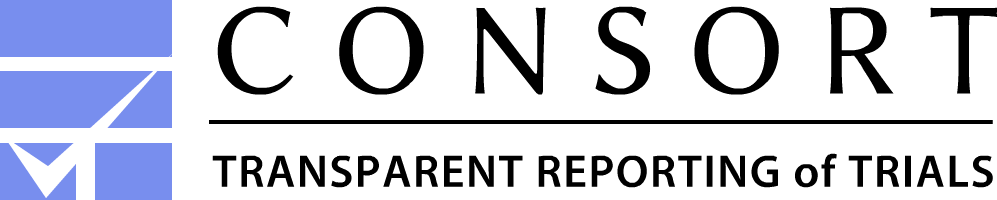
**

**CONSORT 2010 Flow Diagram**

**Allocation**

**Analysis**

**Follow-Up**

**Enrollment**

Assessed for eligibility (n= 35)

Excluded (n= 11)

♦  Not meeting inclusion criteria (n= 5)

♦  Declined to participate (n= 4)

♦  Other reasons (n= 2)

Analysed (n=11)
♦ Excluded from analysis (n=0)

♦ Western blot analysis (n=9), due to muscle tissue shortage.

Lost to follow-up (n=0)

Discontinued intervention (n=1)

- University shutdown due to COVID-19

Allocated to FOR-SUPP (n=12)

♦ Received allocated intervention (n=12)

♦ Did not receive allocated intervention (n=0)

Lost to follow-up (n=0)

Discontinued intervention (n=1)

- University shutdown due to COVID-19

Allocated to PLA-SUPP (n=12)

♦ Received allocated intervention (n=12)

♦ Did not receive allocated intervention (n= 0)

Analysed (n=9)
♦ Excluded from analysis (n=2)

- Poor compliance of wearing knee brace (n=2)

♦ Western blot analysis (n=6), due to muscle tissue shortage.

.

Randomized (n= 24)
