# Supplementary material for: Fortetropin supplementation prevents the rise in circulating myostatin but not disuse-induced muscle atrophy in young men with limb immobilization: A randomized controlled trial
Source: PLoS One. 2023 May 23;18(5):e0286222. doi: 10.1371/journal.pone.0286222 (PMC10204970; doi:10.1371/journal.pone.0286222)

All blots were analyzed using Image Lab version 6.1 (Rio-Rad)

Total protein content for nomalization (**Total form**)

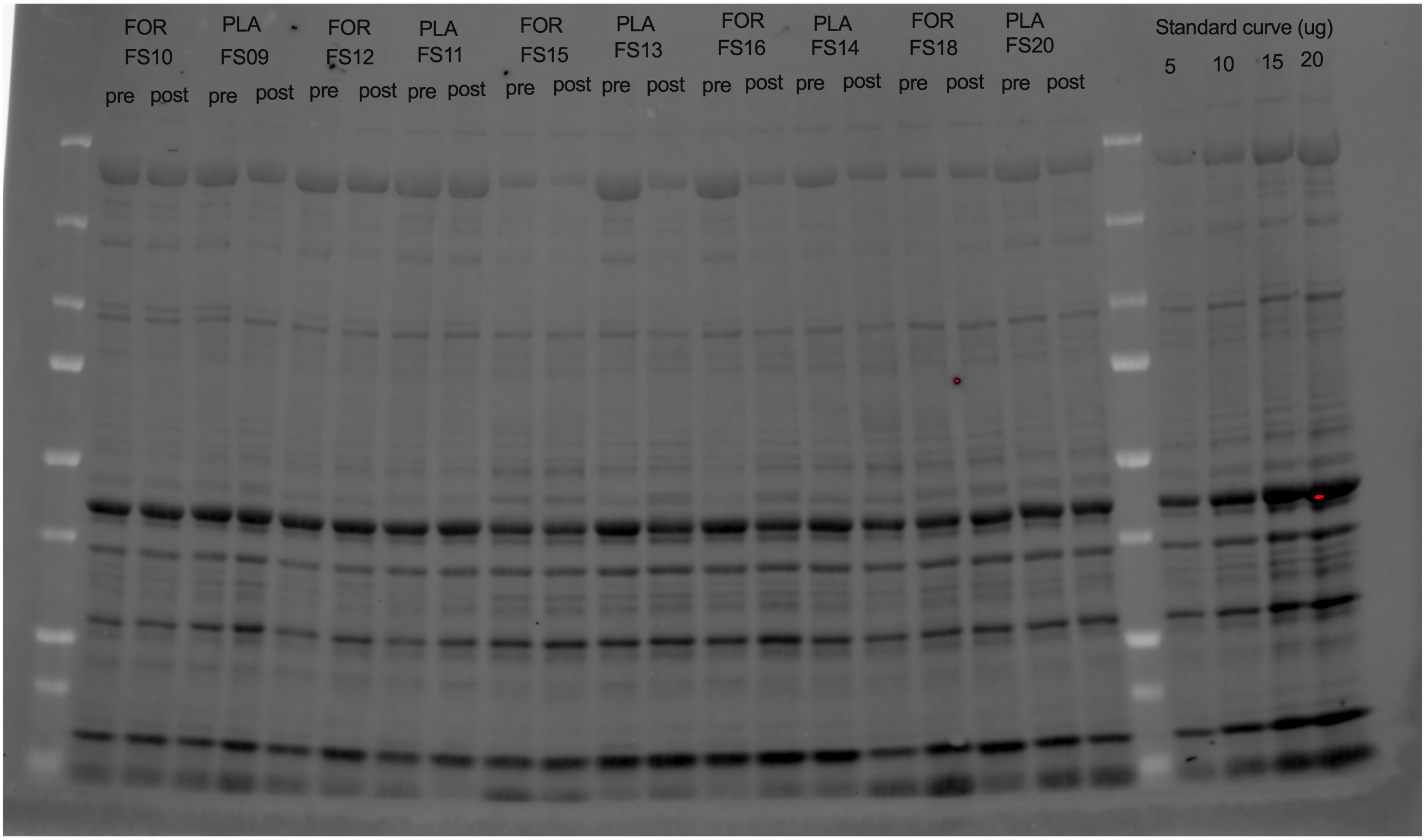

Total mTOR

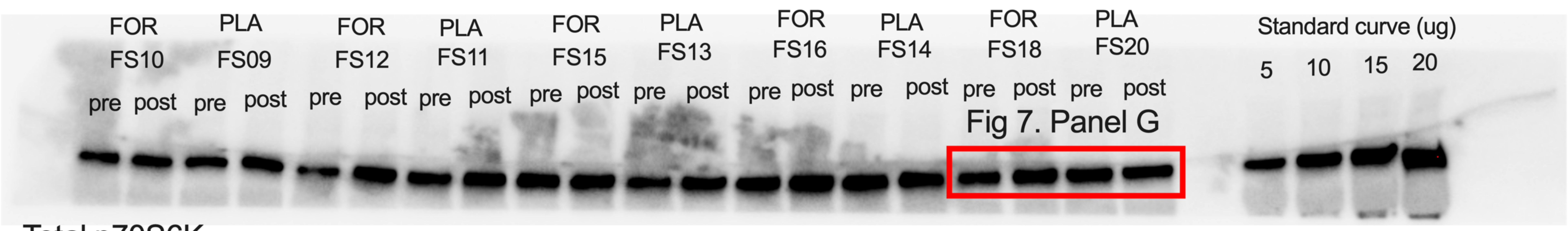

Total p70S6K

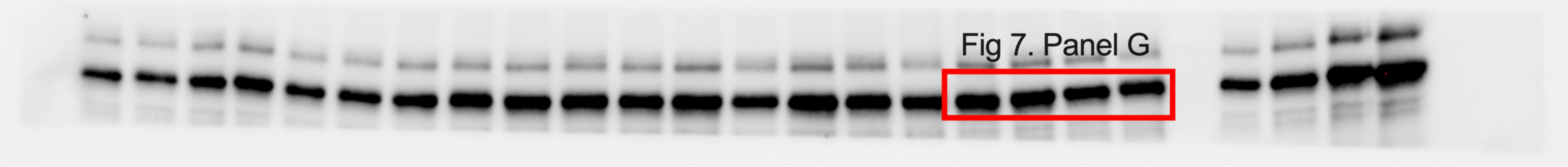

Total S6K

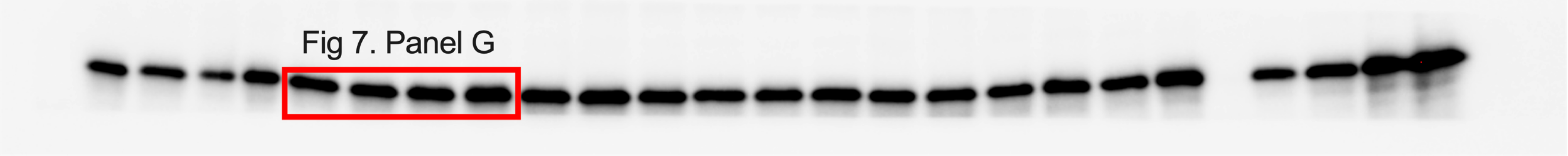

Total 4EBP1

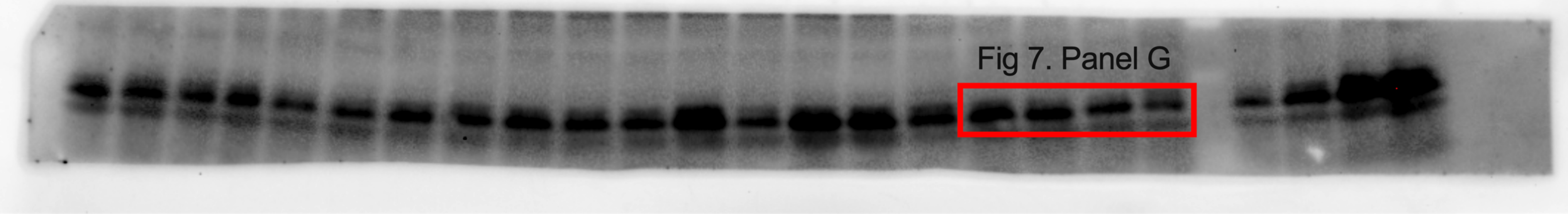

Total protein content for nomalization (**Phospho form**)

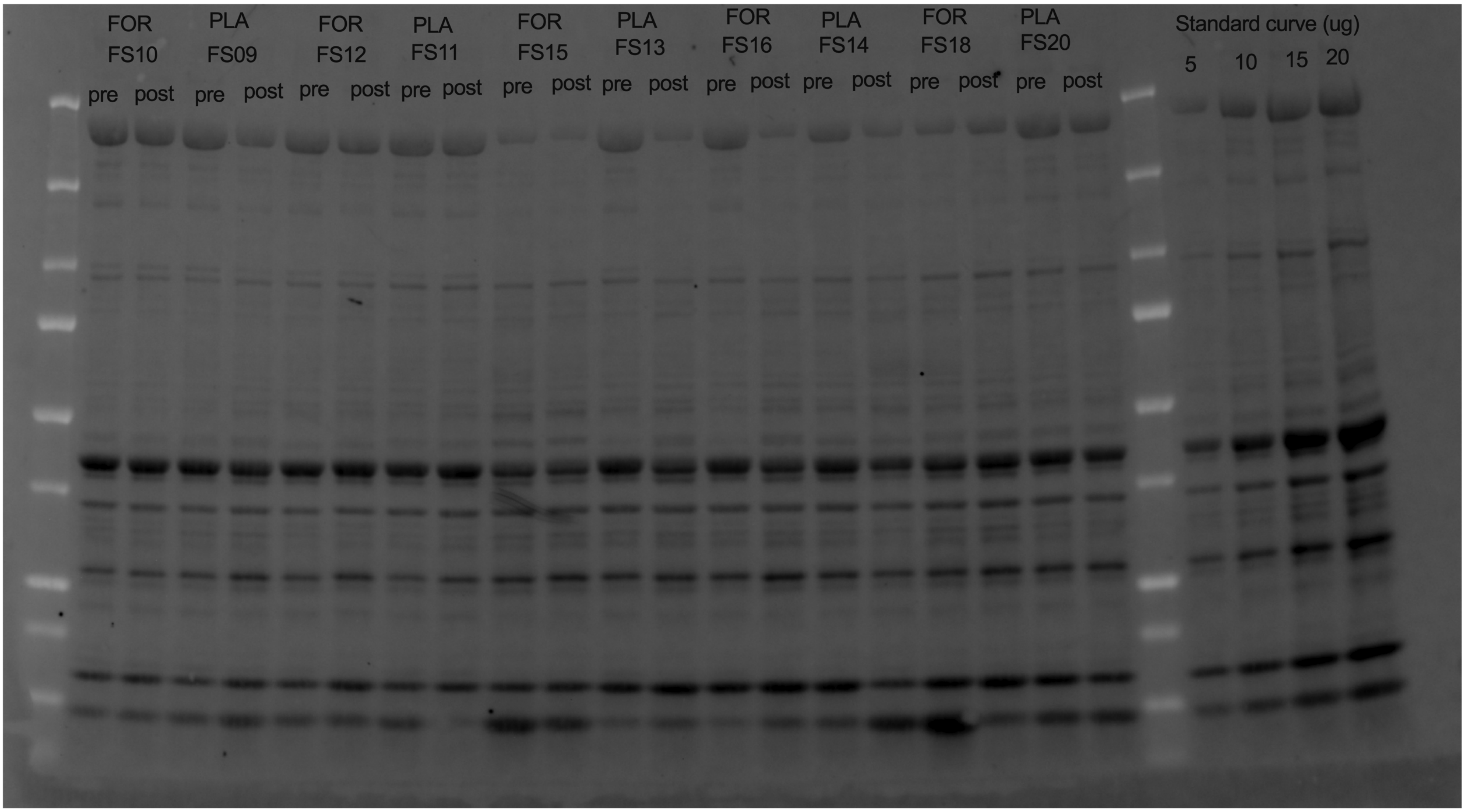

Phospho mTOR

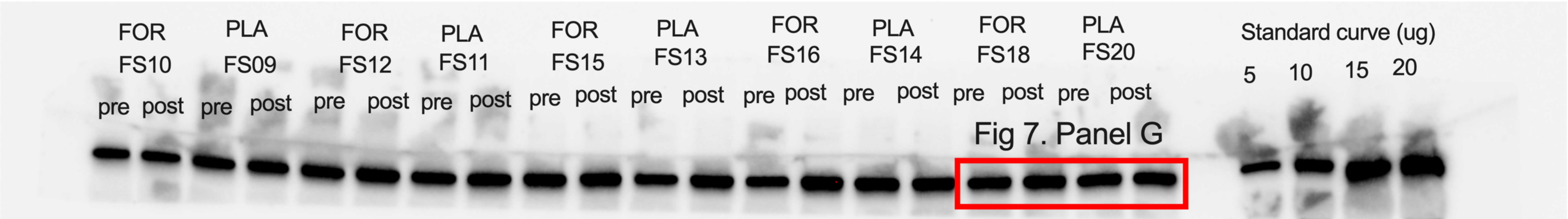

Phospho p70S6K

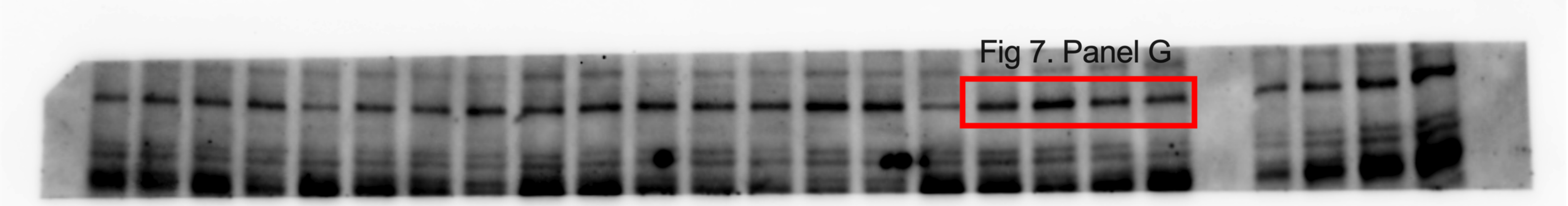

Phospho S6K

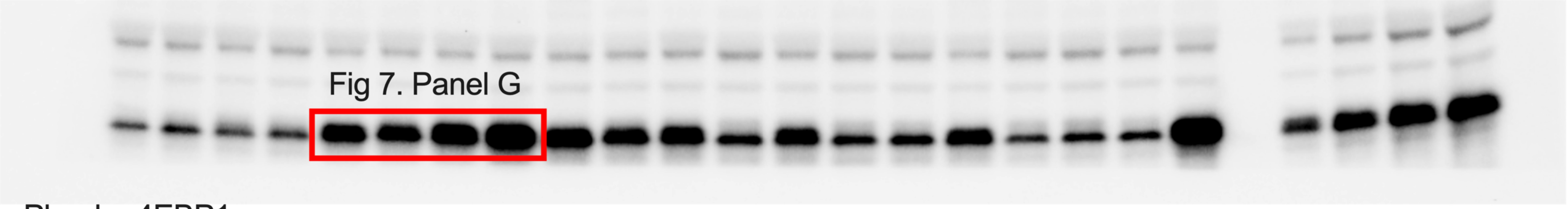

Phopho 4EBP1

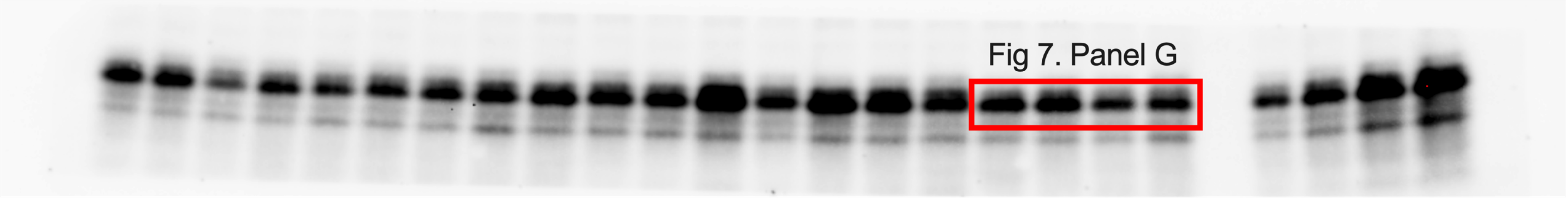

Total protein content for nomalization (**Total form**)

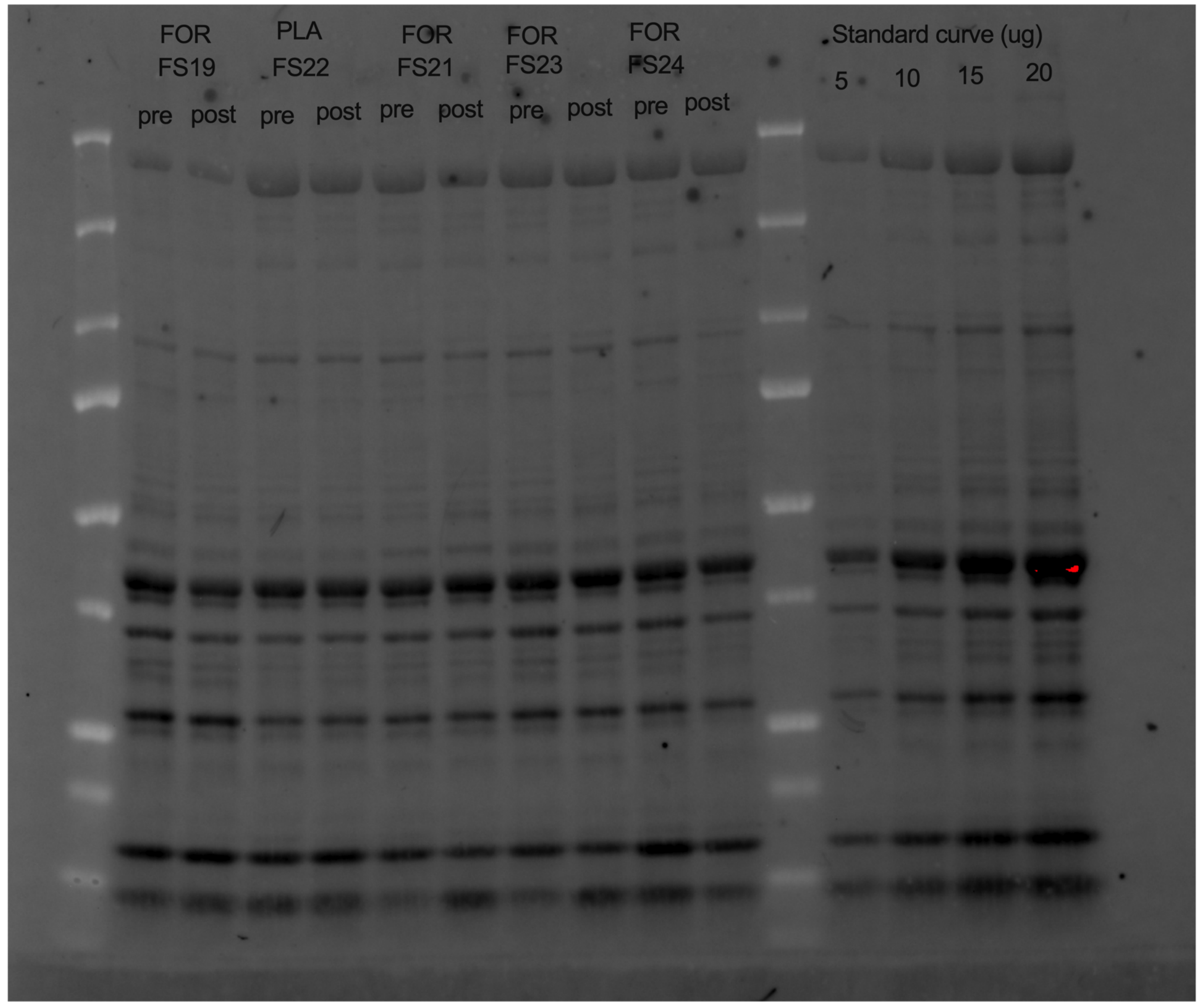

Total mTOR

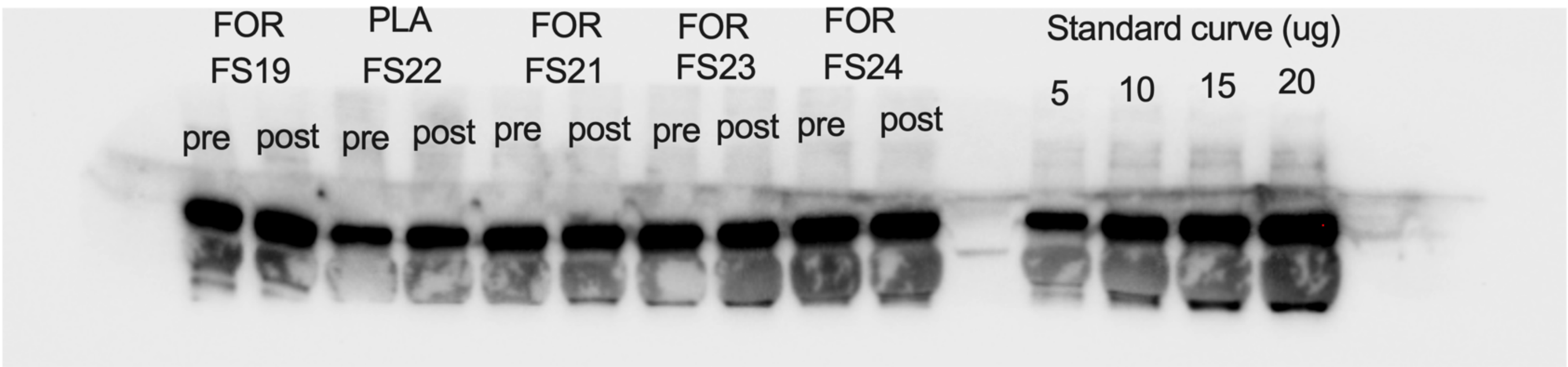

Total p70S6K

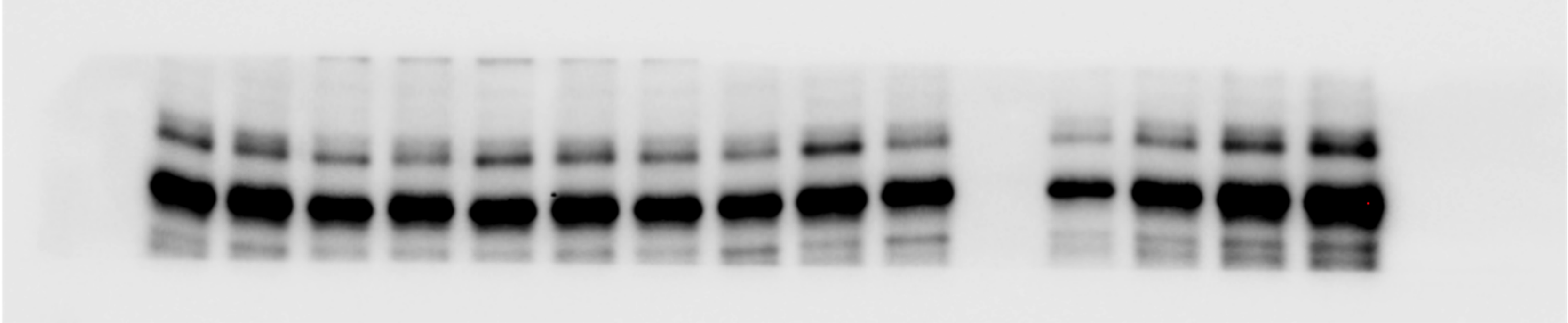

Total S6K

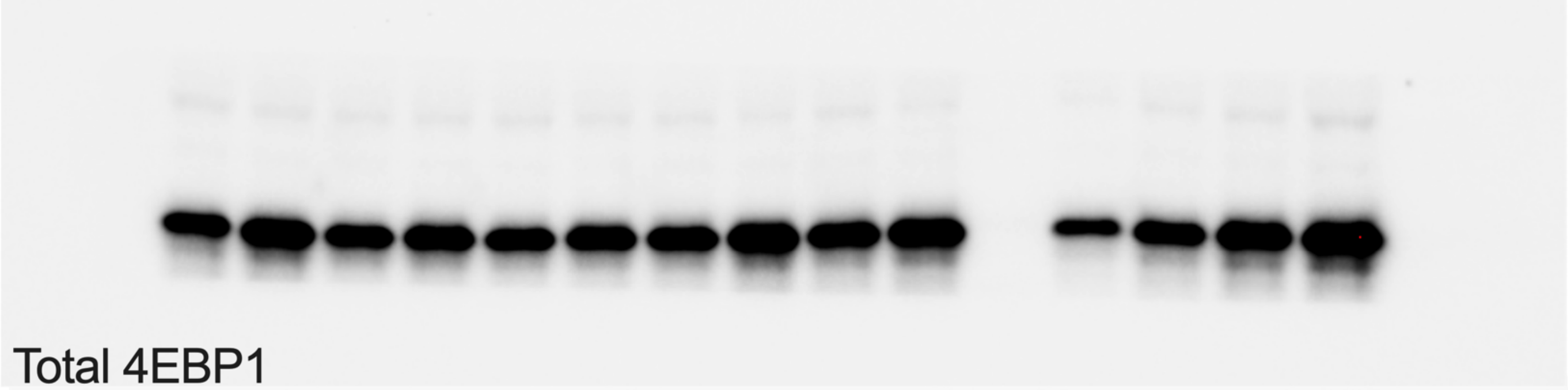

Total 4EBP1

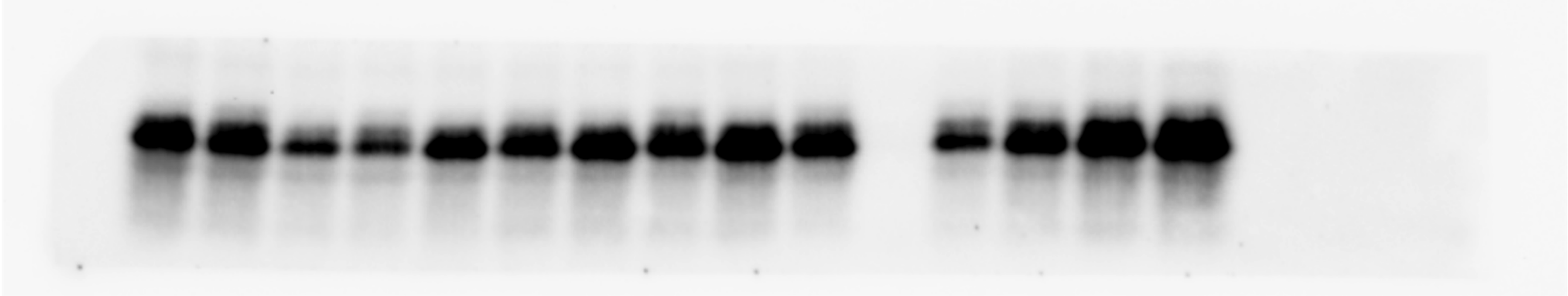

Total protein content for nomalization (**Phospho form**)

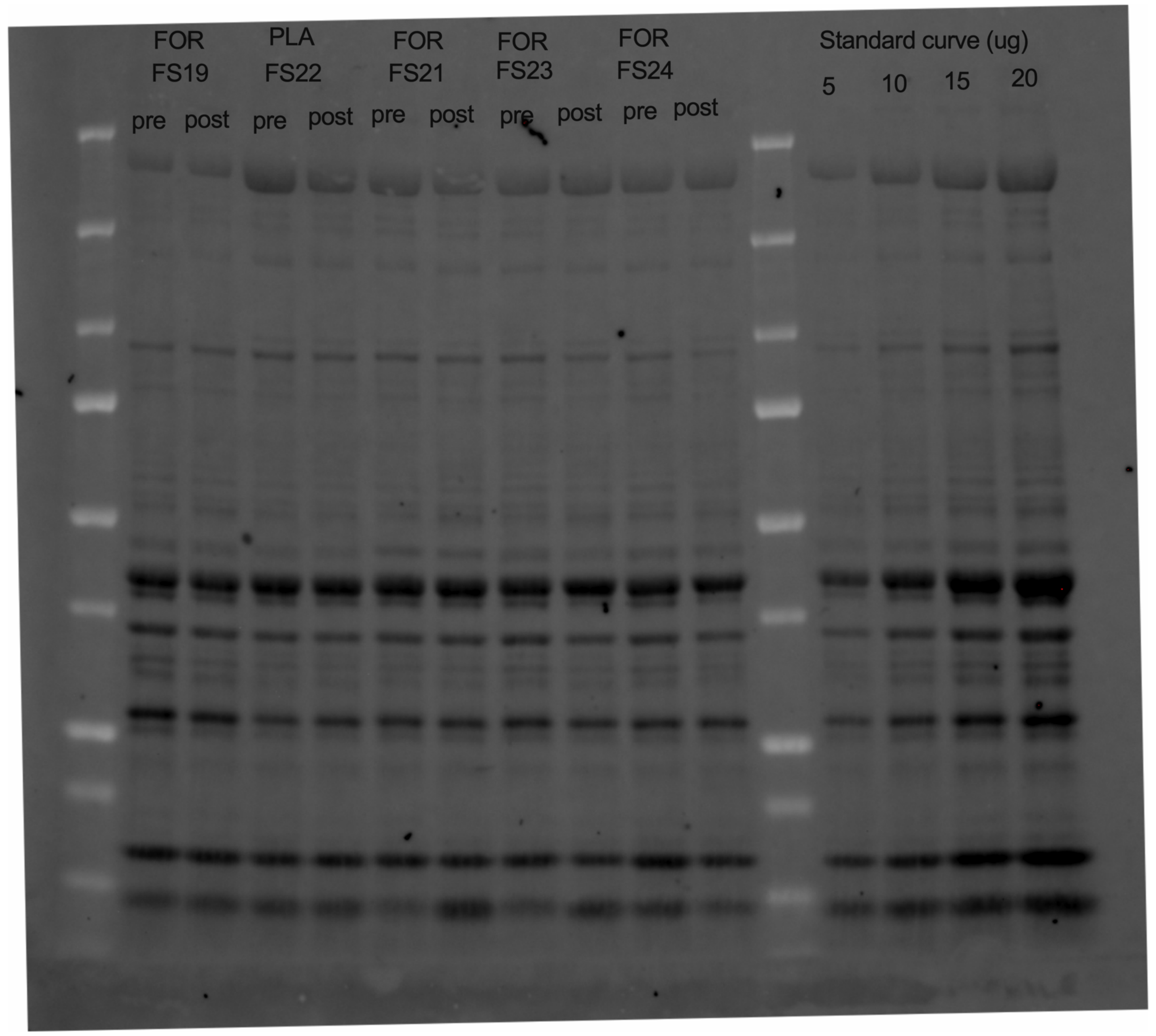

Phospho mTOR

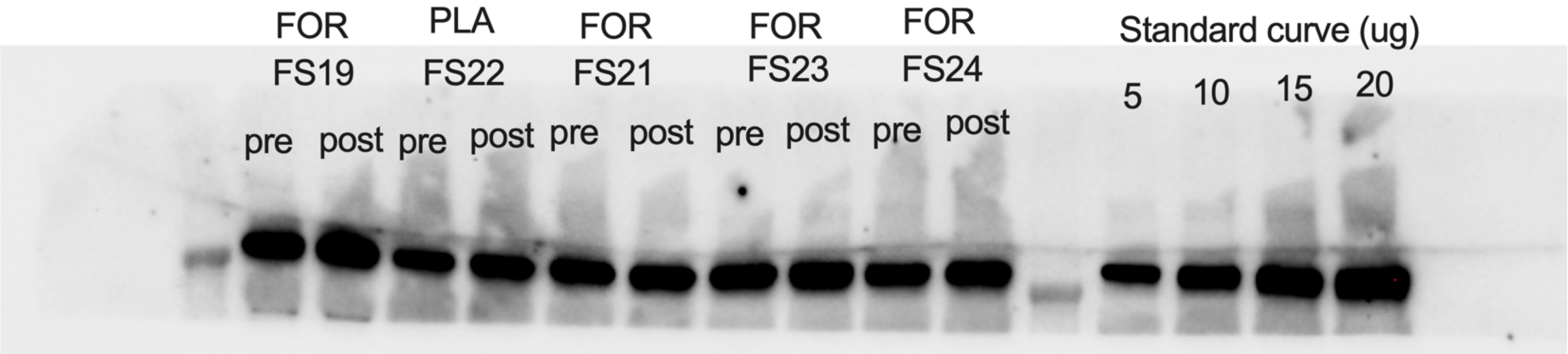

Phospho p70S6K

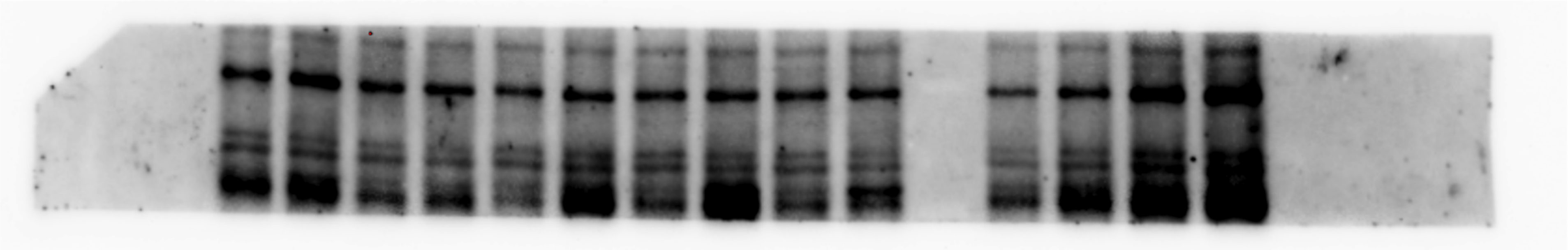

Phospho S6K

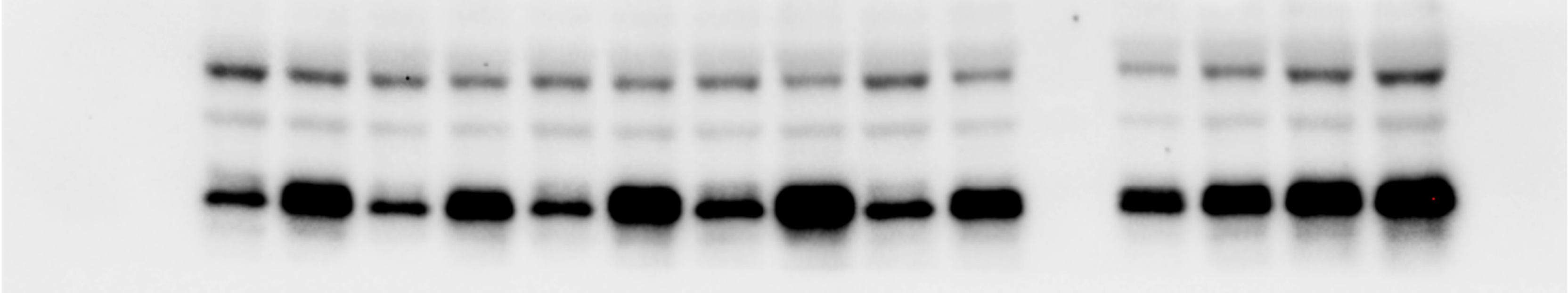

Phospho 4EBP1

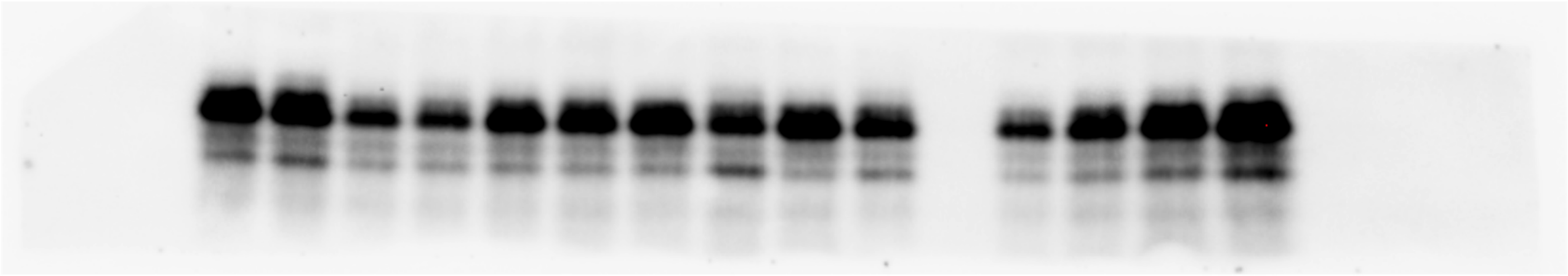

Supplement: S4 File — (PDF) [file pone.0286222.s006.pdf]
