# Supplementary material for: Fortetropin supplementation prevents the rise in circulating myostatin but not disuse-induced muscle atrophy in young men with limb immobilization: A randomized controlled trial
Source: PLoS One. 2023 May 23;18(5):e0286222. doi: 10.1371/journal.pone.0286222 (PMC10204970; doi:10.1371/journal.pone.0286222)
Supplement: Attachment — Submitted filename: PONE-D-22-33716_Responses to Reviewers_R2.pdf [file pone.0286222.s008.pdf]

Thank you for the reviews. We have made revisions to our paper and offer the following respnoses to the reviewers' points.

Reviewer #1: The authors addressed most of my concerns.

In my opinion, the collection of blood samples only on days 14 and 28 should be clearly stated as a limitation of the manuscript.
We have clearly stated that we did not collect blood samples on days 14 and 28 on lines 587-588.

Other minors comments:
L62: it's "Jones et al"
Thank you. We have corrected 'Jone' to "Jones" on line 62.

L537: add please the reference
We have cited the reference on line 540.

Reviewer #2: Minor revisions

1. The reference list is still in mess. It must be improved.
All references are inserted using EndNote software and all are corrected at the typseeting stage of manuscript preparation (by the publisher). Nonetheless, we have corrected the format of reference list according to the PLOS ONE guidelines.

2. The writing of values and units still require correction within the whole manuscript. Apart from '%', Authors should use put space between the value and the unit. Unify the way of writing of units within the whole manuscript and in the figures/tables.
We have corrected this throughout the manuscript and in the figures 2, 3, 4, 5, and 6.

3. Line 62: Put '.' after 'et al.'.
We have put '.' after et al on line 62.

4. What is the rational to use capitals for 'Creatinine', 'Glucose' etc.? – it should be corrected within the whole manuscript.
Thanks for highlighting this. We have corrected the capitals to lower case throughout the manuscript.

5. Lines 140-141: 'All data were stored in a locked filing cabinet and a computer protected 141 by a password' the sentence seems to be not necessary. It can be removed.
We have removed the sentence on line 140.

6. Line 163: Replace 'consumed' with 'intake'.
We have replaced 'conumed' with 'intake' on line 163.

7. Line 225: Replace ‘tissues’ with ‘samples’.
We have replaced ‘tissues’ with ‘samples’ on line 226.

8. Replace ‘minutes’ with ‘min’ within the whole manuscript.
We have replaced ‘minutes’ with ‘min’ throughout the manuscript.

9. Lines 300 – 301: The space between these lines is wider compared to other lines.
We have corrected the space to be same as others by changing the symbol, Δ on line 302.

10. Lines 333 and Table 2: I suggest replacing ‘activity’ with ‘energy expenditure related to/arising from physical activity’ or with similar expression. The expression ‘activity’ is misleading.
We have changed ‘activity’ with ‘energy expenditure by physical activity’ in Table 2 and on lines 335-340.

11. Line 355: ‘The value of immobilized leg LM showed a similar trend as vastus lateralis’ the sentence needs to be reformulated and improved. In the current version it is not clear.
We have removed the sentence to avoid any confusion caused by it on line 359.

12. Line 387: Pleas put the word ‘between’ before ‘groups’.
We have added ‘between’ before ‘groups’ on line 391.

13. Regarding the remark from the first round of the review:
“Lines 337-352: It is not clear to which group (FOR-SUPP or PLA-SUPP) the provided results (CSA, LM, and changes in these indices) refer to? It must be clarified and specified. Are the values means or medians? Thank you. Because there was no time-by-group interaction, we described the data of the main effect for time using the values combined in both groups, with the saying ‘no difference between groups.’ We have revised the sentences to make them clearer for readers on lines 350-362. All data are presented as mean +/- SD.”
Please provide the information that you used mean value from both groups at the beginning of each paragraph within which the procedure was implemented.
We have clearly stated that it’s the mean value of the absolute CSA and immobilization leg LM on lines 353 and 363.

14. Please verify if all the units provided in the Table 3 are correct.
Thanks for highlighting this. We have corrected IU/L to U/L throughout the manuscript.

15. Line 505: Remove ‘There were” from the beginning of the sentence.
Thanks for highlighting this. We have removed ‘There were’ on line 509.

16. Lines 541, 544: Authors can use ‘MPS’ instead of ‘muscle protein synthesis’.
We have revised ‘muscle protein synthesis’ to ‘MPS’ on line 545 and 548.
