# Supplementary material for: Fortetropin supplementation prevents the rise in circulating myostatin but not disuse-induced muscle atrophy in young men with limb immobilization: A randomized controlled trial
Source: PLoS One. 2023 May 23;18(5):e0286222. doi: 10.1371/journal.pone.0286222 (PMC10204970; doi:10.1371/journal.pone.0286222)
Supplement: Attachment — Submitted filename: PONE-D-22-33716_Responses to Reviewers_R1.docx [file pone.0286222.s007.docx]

PONE-D-22-33716

Fortetropin® supplementation prevents the rise in circulating myostatin but not disuse-induced muscle atrophy in young men during immobilization: a randomized controlled trial

**Comments to the Author**

Reviewer #1: This study aimed to investigate the impact of Fortetropin® (FOR) supplementation on immobilization-induced muscle atrophy and weakness. The research question is relevant due to the increasing interest in the scientific literature on disuse countermeasures and the promising findings of FOR supplementation in previous training studies. The authors employed an appropriate disuse model (2-week knee brace immobilization) with subsequent recovery, controlled for/assessed potential confounding factors (diet and physical activity levels) and evaluated alterations in molecular markers involved in muscle atrophy. Overall, the manuscript is clear and easy to follow. However, there are some crucial matters to consider regarding plasma myostatin concentration evaluation.
First, blood samples were collected only on day 1 (onset of the run-in phase) and day 42 (end of the recovery). Due to this limitation in study design, the authors cannot determine with certainty whether the increased myostatin concentration occurred during the immobilization phase or the recovery phase, or both. This is particularly relevant in light of a recent 10-day bed rest study that found an unexpected increase in circulating myostatin after two days of recovery, but not during the unloading period (Oranger et al., 2022). Why did the authors decide to not collect blood samples on day 14 (onset of the immobilization phase) and day 28 (end of the immobilization phase)? Blood sampling is a relatively fast procedure and less invasive compared to muscle biopsies that were obtained also at these two additional time points.

Thank you for highlighting this point. We agreed that collecting blood on days 14 and 28 would bring more information to understand the change in myostatin level. We have adhered to the current protocol for blood draws from the beginning of the research design to minimize the invasive procedures for participants as much as we can. Also, we have thought that the change in myostatin level on muscle mass could be addressed by Pax7+/MSTN+, which is more relevant to skeletal muscle. However, we agree that we do not know the exact time point when the plasma myostatin level increased in PLA-SUPP group in the present study. We ahev revised our manuscript accordingly.

Second, caution is needed in the interpretation of findings exclusively drawn from circulating biomarkers when employing “local” disuse models, such as knee brace immobilization. Indeed, disuse effects are expected mostly at a local muscle level and results obtained from systemic biomarkers may be affected by the relatively low muscle mass undergoing unloading/immobilization (one leg only).

Thank you for highlighting this. We agre that the effects of local disuse are mostly restricted to limb that undergoes disuse. However, single-leg immobilization also significantly reduced overall physical activity, so the model is perhaps more systemic? We have revised our paper to reflect the reviewer’s point.

To overcome these issues, have the authors considered evaluating myostatin transcript and protein levels from muscle biopsies? I strongly recommend performing these additional analyses, if possible.

We had considered performing the analyses the reviewer suggests, but at this point there is tissue remaining from only a sub-sample of the original subjects. Hence, any data we would generate would be from a small sample (3/group) of participants and so unlikely to be useful in interpreting the changes in myostatin.

Other comments:
L1: In the title, the authors suggest that the rise in myostatin occurred during the immobilization phase. However, since blood samples were not collected on days 14 and 28 the authors cannot be completely sure of this (see my main comment).

We have changed ‘during’ to ‘with’ in the title of the revised manuscript.

L28: If word count allows it, I suggest the authors specify that the recovery phase in this study consisted just of the resumption of habitual physical activities.

Thank you for clarifying this point. We have added ‘returning to habitual physical activities’ on line 28.

L34-39: The authors reported just one percentage of reduction and p-value for each muscle force and mass parameter. To which group are they referred (FOR-SUPP or PLA-SUPP)? Or is it an average?

The percentage values and the p-values (of the percentage values) show the main effect for time of the *vastus* *lateralis* CSA, LM, and isometric peak torque combined in both groups after detecting no interaction from two-way ANOVA (group x time).

L39-40: As for the title, I believe the authors cannot affirm that the rise in myostatin is due to the immobilization period.

Thank you. We have changed ‘during’ to ‘with’ on line 41.

L59: I feel some background on the role of myostatin in disuse atrophy is needed here in the introduction. Is increased myostatin an established finding with muscle disuse/immobilization? To the best of my knowledge, there is conflicting evidence on the effect of human limb disuse/immobilization on myostatin levels. Previous studies employing cast immobilization found unchanged (Jones et al., 2004; Chen et al., 2007) or increased (Dirks et al., 2014) MSNT expression, or even decreased myostatin protein content (Snijders et al., 2014). Increased myostatin gene and protein levels were observed in response to 3 days of unilateral lower limb suspension (ULLS) (Gustafsson et al., 2010), but no changes were found in a longer 14-day ULLS (de Boer et al., 2007). Finally, 25 days (Zachwieja et al., 1999), but not 10 days (Oranger et al., 2022) of bed rest increased circulating myostatin levels. Results in animal disuse studies seem also ambiguous (Carlson et al., 1999; Wehling et al., 2000; Allen et al., 2009). I believe the authors should cite and discuss at least part of this previous literature in the manuscript to give to the reader a broader background.

We thank the reviewer for commenting on this point. We have discussed the change in myostatin level in the single-leg immobilization model on lines 59 – 64.

L82: Authors should add DXA procedures to the Methods section or at least refer the reader to previous studies using the same procedures.

Thank you. We have cited a previous study that used DXA for the same procedures and purpose as the present study on line 134.

L145: How was the amount of daily FOR supplementation (19.8g/d) determined?

Based on the previous study (PMID:27333407) showing the reduced myostatin level in plasma after consuming 19.8g/d, that was used as the dose for the present study.

L168: Were the subjects asked to refrain from intense physical activity during the whole protocol (run-in + immobilization + recovery) or just during the immobilization phase?

Yes, the subject refrained from intense physical activity during the whole protocol.

L180-181: Are the ultrasound-derived CSA values presented in the article the average of these 7-9 images? Why did you choose to collect the images at 2cm intervals, instead of capturing different muscle regions (e.g. 70%, 60%, 50%, 40% 30%, 20% of femur length) to take into account potential regional differences?

Depending on the size of the quadriceps of participants, we needed to take 7-9 images on a region (50% of the distance between the top of the patellar bone and the greater trochanter of the femur) in a longitudinal of the *vastus lateralis* to get one intact CSA image. The 7-9 images were taken at a 2cm interval, and an entire CSA image of vastus lateralis was acquired by stitching these images together. The ultrasound probe that takes the images is 4cm long. So, the 2cm intervals provide 2cm of the overlap region with an adjacent image, and the overlap regions were used to align subcutaneous fat, superficial and deep aponeuroses, and intramuscular fat for stitching the images.

L210: Could you please provide more information regarding muscle biopsies exact location and precautions to avoid the effects of pre-sampling?

Thanks for clarifying this point. We have added this information on lines 220-222.

L313: How was participants’ compliance evaluated?

We locked the buckle of the knee brace using zip ties on which a unique signature was written. The participant could not remove the knee brace without destroying the zip tie and signature. Thus, if the zip tie got damaged or the signature disappeared, we could easily judge the participant’s compliance.

L332: Have the authors collected any functional or morphological data on the control leg? I think it would be a nice addition to the manuscript.

Thank you for the comment. Unfortunately, we did not collect data from the contralateral leg.

L364: I suggest the authors state here that no differences were observed in absolute CSA values of Type I fibres, as reported in Figure 3A.

We have added ‘absolute’ in front of CSA to clarify on lines 380 and 386.

L454: The authors are invited to report also in the main text that WB was performed only on a subgroup of participants (N=9 for FOR-SUPP and n=6 for PLA-SUPP) due to shortage of muscle tissue.

Thank you for noting this point. We have added this statement on lines 277-279.

L455: Add “(data not shown)” after “E3 ubiquitin ligases”, as this result is not reported in any figures or tables.

We reported the result of Atrogin-1 and MuRF1, which are the best characterized E3 ubiquitin ligases, as E3 ubiquitin ligases.

L480: Did E3 ubiquitin ligases change their expression with immobilization? In the results section (L455), the authors reported only that no interactions between group and time for E3 ubiquitin ligases were found, but here in the discussion, the authors suggest that is one of the proved molecular mechanisms involved in muscle atrophy with disuse.

Thank you. We mentioned the E3 ligases result to support no effect of Fortetropin in molecular mechanisms at skeletal muscle level between groups, not for disuse atrophy. E3 ligases (Atrogin-1 and MuRF1) showed no changes with immobilization.

L486: I suggest the authors discuss here that the literature however is not always in agreement and different studies found unchanged or decreased myostatin levels in disuse scenarios (Jones et al., 2004; de Boer et al., 2007; Snijders et al., 2014; Oranger et al., 2022).

Thank you for highlighting this point. We have added the statement on lines 509-511.

L507: While MuRF1 and Atrogin-1 expressions did not differ between conditions, they were also unaltered by immobilization, thus I am not sure these results are in line with the reduced muscle size and LM observed.

Thanks for noting this point. Unlike the rodent model, previous studies demonstrate the dominant mechanism causing disuse muscle atrophy is decreased protein synthesis in humans rather than increased protein degradation. This cross-talk (PMID:25512435) covers the idea. Also, the transcripts analyzed at each point before and after immobilization, which is the snapshot outcome, often do not reflect the changes induced over (throughout) the duration of intervention – see also PMID: 35606155 and 34342902.

L510-511: Did the authors expect this increase in myostatin-positive SC with immobilization? I believe this novel finding should be further discussed.

Thank you. We have added more discussion about the myostatin-positive SC on lines 536-540.

L543: The authors forgot to add “complexity” after “motor unit potential”. In addition, I suggest specifying that the retraining period of the cited study was based on resistance training, differently from the present manuscript.

Thank you for highlighting it. We have added ‘complexity’ after ‘motor unit potential’ on line 573 and have specified the way of recovery on line 575.

L614: please fix reference number 12, probably an error with the reference manager occurred.
Thanks for noting this error. Yes, there was an error with the Endnote, the reference manager software. We have corrected them in the revised manuscript.

References:
- Allen DL, Bandstra ER, Harrison BC, Thorng S, Stodieck LS, Kostenuik PJ, Morony S, Lacey DL, Hammond TG, Leinwand LL, Argraves WS, Bateman TA & Barth JL (2009). Effects of spaceflight on murine skeletal muscle gene expression. J Appl Physiol 106, 582–592.
- de Boer MD, Selby A, Atherton P, Smith K, Seynnes OR, Maganaris CN, Maffulli N, Movin T, Narici M V. & Rennie MJ (2007). The temporal responses of protein synthesis, gene expression and cell signalling in human quadriceps muscle and patellar tendon to disuse. J Physiol 585, 241–251.
- Carlson CJ, Booth FW & Gordon SE (1999). Skeletal muscle myostatin mRNA expression is fiber-type specific and increases during hindlimb unloading. Am J Physiol - Regul Integr Comp Physiol 277, 601–606.
- Chen YW, Gregory CM, Scarborough MT, Shi R, Walter GA & Vandenborne K (2007). Transcriptional pathways associated with skeletal muscle disuse atrophy in humans. Physiol Genomics 31, 510–520.
- Dirks ML, Wall BT, Snijders T, Ottenbros CLP, Verdijk LB & Van Loon LJC (2014). Neuromuscular electrical stimulation prevents muscle disuse atrophy during leg immobilization in humans. Acta Physiol 210, 628–641.
- Gustafsson T, Osterlund T, Flanagan JN, Von Waldén F, Trappe TA, Linnehan RM & Tesch PA (2010). Effects of 3 days unloading on molecular regulators of muscle size in humans. J Appl Physiol 109, 721–727.
- Jones SW, Hill RJ, Krasney PA, O’Conner B, Peirce N & Greenhaff PL (2004). Regulation of skeletal muscle mass and adipose tissue mass by follistatin and follistatin-related gene (FLRG) and development of novel polypeptides as medical drugs. FASEB J; DOI: 10.1096/fj.03-1228fje.
- Oranger A, Storlino G, Dicarlo M, Zerlotin R, Pignataro P, Sanesi L, Narici M, Pišot R, Simunič B, Colaianni G, Grano M & Colucci S (2022). Impact of 10-day bed rest on serum levels of irisin and markers of musculoskeletal metabolism. FASEB J 37, e22668.
- Snijders T, Wall BT, Dirks ML, Senden JMG, Hartgens F, Dolmans J, Losen M, Verdijk LB & Van Loon LJC (2014). Muscle disuse atrophy is not accompanied by changes in skeletal muscle satellite cell content. Clin Sci 126, 557–566.
- Wehling M, Cai B & TidballL J (2000). Modulation of myostatin expression during modified muscle use. FASEB J 14, 103–110.
- Zachwieja JJ, Smith SR, Sinha-Hikim I, Gonzalez-Cadavid N & Bhasin S (1999). Plasma myostatin-immunoreactive protein is increased after prolonged bed rest with low-dose T3 administration. J Gravitational Physiol.

We thank the reviewer for offering the relevant references.

Reviewer #2: Major revisions
1. Sample size determination:
a) Line 104: please provide the references for the effect size interpretation;

Thank you for noting this. We have cited a reference (PMID:23997866) for the effect size on line 109.

b) Line 105: it is indicated that total calculated sample size is 24 (12 participants per each group). While, the final statistical analysis covered 11 participants is FOR-SUPP group and 9 participants in PLA-SUPP groups. In this circumferences, what were the actual effect size, alpha and power? Was the final sample size sufficient? Why Authors did not accounted for possible drop outs at the stage of a priori sample size determination? Western blot analysis was performed for even lower number of participants.

Thank you. Statistical power in the final sample size has lowered to 75%, with the effect size and alpha unchanged. We acknowledge the reduced power of our analysis due to the insufficient sample size caused by unavoidable circumstances (COVID-19 and compliance with wearing a knee brace). Thus, we mentioned the limitation on lines 585-587. Also, the global COVID-19 pandemic halted the present study (in-person human research) for approximately 18 months. It substantially impacted the full recruitment of the present study due to time constraints. Nonetheless, we think the present study provides novel human model data that contributes to our understanding of the effect of the supplements and a disuse event on human skeletal muscle.

2. What was the reason for performing blood evaluations ‘solely’ at days 1 and 42? Why were they not performed at days 14 and 28?

Thank you for highlighting this point. We have adhered to this plan for blood draws from the beginning of the research design to minimize the invasive procedures for participants as much as possible because we have thought that the change in myostatin level on muscle mass could be addressed by Pax7+/MSTN+, which is more relevant to skeletal muscle. However, we agreed that collecting blood on days 14 and 28 would bring more information.

3. Lines 143-145: how was the nutritional value of Fortetropin supplement evaluated? Please provide methodology and/or relevant references.

Thank you. We have added more information about the Fortetropin analysis on lines 152-154.

4. Line 161: With respect to protein intake during the immobilization phase (1.2 g protein per kg of body mass), Authors are referring to the paper published in 2022 (doi: 10.1002/jcsm.12922), while the study on Fortetropin supplementation started in 2020. Please provide the references/guidelines that actually were implemented when establishing protein intake during immobilization phase.

Thanks for noting this. We have added a reference (PMID:29635313) supporting the rationale of the protein dose in young adults on line 170.

5. It was mentioned that energy requirements during immobilization phase were calculated by multiplying basal metabolic rate (as calculated with Harris-Benedict equation) by physical activity level. Further Authors indicated that energy expenditures were monitored using Actigraph wGT2X-BT activity monitor. Thus, is it possible to compare the calculated/pre-assumed energy expenditures and the actual/established with Actigraph energy expenditures? What was the actual energy balance in participants during immobilization phase?

Thanks for highlighting this. We have revised energy expenditure, which was miswritten, to physical activity on lines 177-178. We analyzed physical activity level (kcal/d), not whole energy expenditure, during each protocol phase. So, unfortunately, we could not compare actual energy expenditures and pre-assumed ones.

6. Lines 337-352: It is not clear to which group (FOR-SUPP or PLA-SUPP) the provided results (CSA, LM, and changes in these indices) refer to? It must be clarified and specified. Are the values means or medians?

Thank you. Because there was no time-by-group interaction, we described the data of the main effect for time using the values combined in both groups, with the saying ‘no difference between groups.’ We have revised the sentences to make them clearer for readers on lines 350-362. All data are presented as mean +/- SD.

7. Lines 362-374: It is not clear to which group (FOR-SUPP or PLA-SUPP) the provided results (muscle fiber CSA) refer to? It must be clarified and specified. Although, there were no significant differences between groups, still it is impossible that the results are exactly the same in both groups. Maybe be it would be reasonable to decline from providing the exact mean results/values in the text (leave ‘P’ values only) – while the exact results are provided in the corresponding figures. The figures are very clear and highly-informative.

Thanks for highlighting this. As mentioned above, the data are combined in both groups to describe the main effect of time. We have revised our manuscript to clarify this point on lines 381-388.

8. Please refer point 7 to results on isometric knee-extensor torque.
Thank you. It is also the same reason, describing the main effect for time. We have revised it to make it clear on lines 406-407.

Minor revisions
1. Initial concentrations of plasma myostatin were 5487 ± 489 pg/mL in FOR-SUPP group and 4221 ± 541 pg/mL in PLA-SUPP group. Thus, was there a significant difference in baseline plasma myostation between groups? Please correct decimal places in lines 409-410 (results section)

Thanks for noting this point. There was no difference in myostatin level in plasma between FOR-SUPP and PLA-SUPP (*P*=0.104). We have rounded the decimal up or down to the whole number on lines 428-429.

2. Please standardize the way of recording of ‘Fortetropin’ name throughout the manuscript – standardize to use or not to use ‘®’ sign.

Thank you. We have standardized the expression of Fortetropin to Fortetropin®.

3. Line 40: the following sing ‘-‘ is lacking the writing of ‘2-wk’.

Thank you. We have added ‘-‘ between 2 and w on line 41.

4. Please standardize using or not to using spaces between special signs such as ‘=/±/>/5. Please standardize the way of recording of ‘vs.’ or ‘vs’ throughout the manuscript.

Thanks for highlighting this. We have standardized the things the reviewer noted throughout the manuscript.

6. Line 108-111: In general, this part of the text is unclear and needs to be reworded.

Thank you. We have rewritten it on lines 114-117.

7. Line 119, 471: Consider not using first person expressions.

We have revised the first-person expressions to passive voice on line 126 and 492.

8. Line 126: Precise the writing of the hours of arriving to the laboratory.

We have re-written the time to 08:00 on line 133.

9. Line 166: Correct the writing of word ‘exercise’.

Thanks for noting this. We have corrected it on line 175.

10. Standardize the writing of the names of study phases throughout the manuscript – sometimes capitals are used as a first letter of the name of the phases, and in the other parts of the manuscript lowercase is used.

Thanks for highlighting it. We have standardized the name of phases throughout the manuscript.

11. Lines 168-169: Correct and standardize the writing of units (i.e., ‘steps/d-1; energy expenditure, kcal/d; metabolic equivalents of task [METs/d-1]’). It also refers to Table 2.

We have corrected and standardized the units on lines 177-178 and Table 2.

12. Line 194: Maybe it is better to write ‘3 trials’ if you are using the numbers in the earlier part of the paragraph (i.e., 4 repetitions).

We have rewritten ‘three’ to ‘3’ on line 203.

13. Lines 204-205: What is the rational to use capitals for ‘Creatinine’, ‘Glucose’ etc.?

Thank you. We have changed the capitals to lowercase.

14. Line 306-307: Part of results are expressed as median and percentile ranges.

All the values are expressed as mean ± SD in the manuscript. Only the figures presented using the box and whiskers show the median and the 25^th^ and 75^th^ percentile as well as the mean to give more information to readers. We indicated it in legend of each figure.

15. The references list it messy. It must be corrected according to PLoS One guidelines.

Thanks for noting this. We did not notice the error occurred by Endnote, a reference manager software. We have corrected the reference list.

16. Please consider emergence ‘Conclusions’ from ‘Discussion section’.

We have made the Conclusions section following the Discussion on line 589.
